# Which medical error to disclose to patients and by whom? Public preference and perceptions of norm and current practice

**DOI:** 10.1186/1472-6939-11-17

**Published:** 2010-10-18

**Authors:** Muhammad M Hammami, Sahar Attalah, Mohammad Al Qadire

**Affiliations:** 1 Centre for Clinical Studies and Empirical Ethics King Faisal Specialist Hospital and Research Centre Riyadh, Saudi Arabia

## Abstract

**Background:**

Disclosure of near miss medical error (ME) and who should disclose ME to patients continue to be controversial. Further, available recommendations on disclosure of ME have emerged largely in Western culture; their suitability to Islamic/Arabic culture is not known.

**Methods:**

We surveyed 902 individuals attending the outpatient's clinics of a tertiary care hospital in Saudi Arabia. Personal preference and perceptions of norm and current practice regarding which ME to be disclosed (5 options: don't disclose; disclose if associated with major, moderate, or minor harm; disclose near miss) and by whom (6 options: any employee, any physician, at-fault-physician, manager of at-fault-physician, medical director, or chief executive director) were explored.

**Results:**

Mean (SD) age of respondents was 33.9 (10) year, 47% were males, 90% Saudis, 37% patients, 49% employed, and 61% with college or higher education. The percentage (95% confidence interval) of respondents who preferred to be informed of harmful ME, of near miss ME, or by at-fault physician were 60.0% (56.8 to 63.2), 35.5% (32.4 to 38.6), and 59.7% (56.5 to 63.0), respectively. Respectively, 68.2% (65.2 to 71.2) and 17.3% (14.7 to 19.8) believed that as currently practiced, harmful ME and near miss ME are disclosed, and 34.0% (30.7 to 37.4) that ME are disclosed by at-fault-physician. Distributions of perception of norm and preference were similar but significantly different from the distribution of perception of current practice (P < 0.001). In a forward stepwise regression analysis, older age, female gender, and being healthy predicted preference of disclosure of near miss ME, while younger age and male gender predicted preference of no-disclosure of ME. Female gender also predicted preferring disclosure by the at-fault-physician.

**Conclusions:**

We conclude that: 1) there is a considerable diversity in preferences and perceptions of norm and current practice among respondents regarding which ME to be disclosed and by whom, 2) Distributions of preference and perception of norm were similar but significantly different from the distribution of perception of current practice, 3) most respondents preferred to be informed of ME and by at-fault physician, and 4) one third of respondents preferred to be informed of near-miss ME, with a higher percentage among females, older, and healthy individuals.

## Background

In healthcare, it is not uncommon that patients are exposed to risks of harm. Some risks are predictable, at least at statistical level, and an informed consent is obtained. Other risks, such as those occurring because of medical errors (ME) are in a sense unpredictable and an informed consent can not be obtained. An ME is defined as an act or omission that would have been judged wrong by knowledgeable peers at the time it occurred [1]. Some ME may not materialize into harm; a near miss is an event that under slightly different circumstances could have been an accident, either because the error was detected and corrected in time or because the patient was just lucky [2]. When an ME occurs, two actions should be considered: reporting it to the healthcare system (and through it to potential future patients) and disclosing it to the patient involved. Reporting ME is paramount for quality and safety improvement and should include near miss ME [1,3]; compared to reporting harmful ME, reporting near miss ME offers greater frequency and fewer barriers to data collection [3].

Full disclosure of ME includes an explicit statement that an error (rather than just a "complication") occurred, basic description of the error, who committed the error, why it did happen, how recurrences will be prevented, and an apology [4,5]. Non disclosure of harmful ME is considered a violation of ethical principles from both deontological and consequentialist perspectives [6]. A policy of open disclosure standard that demands disclosure of critical events by the provider or the institution [7] was promulgated in 2001 by the Joint Commission on Accreditation of Healthcare Organizations (JCAHO) and is now reflected in similar initiatives in the UK, Canada, and Australia [8-12]. These regulations and theoretical ethical considerations are consistent with the results of empirical ethics studies in Western cultures showing that patients overwhelmingly desire full disclosure of harmful ME [5,13-17] and that full disclosure is likely to have a positive or no effect on how patients respond to ME [18].

Disclosure of near miss ME to patients is a matter of controversy [19,20] and an issue on which current guidelines are silent. Disclosure is recommended by some [21-24] but not all authors in the field [2,25]. The American Society for Reproductive Medicine states that if there is clearly no adverse effect of a ME, disclosure may not be obligatory if it may unnecessarily increase patient's stress [26]. In the few empirical studies that have specifically addressed near miss ME, 88% to 92% of patients desired disclosure [15,27]. In contrast, most physicians opposed near miss ME disclosure [5]. Determining who should disclose ME is another matter of controversy. According to Liang model [21] and the policy described by Kraman and colleagues [28], risk management committee should be responsible for disclosure. Others believe that the responsibility for disclosure belongs to the physician [10,11,22,29]. JCAHO standard requires that a responsible licensed independent practitioner or his/her designee explains the outcome [7].

Current disclosure literature contains important but unanswered questions such as how patient' preferences for disclosure vary along cultural and other dimensions [10,11]. An individual's ethical decision-making is based on his/her values and beliefs. Although major ethical values are rather universal, ethical values are subject to individual interpretation and people naturally differ in their values' hierarchy and in their beliefs. Autonomy is placed at the top of the "moral mountain" and is given a "place of honor" in Western but not other ethics [30]. Further, there are several meanings of autonomy along a spectrum from a negative or anti paternalistic model to a positive mandating model [31]. Furthermore, it has been argued that respect should be for the person rather than purely for autonomy [31,32]; trust in providers, treatment with respect, and dignity were more closely associated with patients overall evaluation of their hospitals than adequate involvement in decision-making [33]. To our knowledge, there is no study on patients' views on disclosure of ME that has been conducted in Arabic/Islamic countries or that compared preference (a statement about the person who has the preference) and perception of norm (a statement about the thing which is being judged).

The aim of this study was to obtain empirical evidence on public views on disclosure of ME in the outpatient's setting at a tertiary care hospital in Saudi Arabia. We examined preference, perception of norm, and perception of current practice on two topics, which ME to be disclosed and who to disclose ME.

## Methods

This cross sectional survey was conducted in accordance with the ethical principles contained in the Declaration of Helsinki and after approval of the Research Ethics Committee of the King Faisal Specialist Hospital and Research Center (KFSH&RC) in the period from November 2007 to March 2009. All respondents gave verbal consent.

Two sets of three questionnaires addressing personal preference, perception of norm (what is appropriate in general/should be done), and perception of current practice at KFSH&RC regarding which medical error is disclosed to patients (set one) and by whom (set two) were developed by the authors in Arabic language based on literature review. After initial development, the questionnaires were presented for comments to 6 physicians and revised accordingly (minor changes in language usage to have consistency throughout the questionnaires). Face validity was assessed by interviewing 10 respondents after completing the questionnaires. The final version was pilot tested on 10 other individuals for clarity and stability (2-3 days) and found suitable. An English translation (accuracy confirmed by back translation) of the two questionnaires on personal preference is shown in Table 1. Similar statements with appropriate modifications were used for the questionnaires on perceptions of norm and current practice. For example, we used the phrase "I prefer" combined with "to be" and "my/me" to indicate personal preference and "I think" combined with "should be" and "patient/his" to indicate perception of norm. For perception of current practice questionnaires, "I prefer" was omitted and "is" was combined with "patient/his". The statements in each questionnaire were arranged from least to most demanding. Before completing the questionnaires, participants were given the following introductory information on medical errors: "Clinical practice, just like any other beneficial practice, could hardly be completely free from harm. Such harm can be divided into two types: 1) harm that can be predicted and thus can be avoided, e.g. anaphylactic shock caused by penicillin administration to a person known to have penicillin allergy, and 2) harm that can't be predicted/avoided, e.g. inflammation of the bowel after some antibiotics treatment. The first type is called medical error. A medical error is defined as failure to complete a planned medical action as intended, or the use of a wrong plan to achieve an aim. Medical errors may or may not cause harm, for example, penicillin could be wrongly prescribed by a physician but not given to the patient because the error is discovered and corrected in time by a pharmacist or nurse. Physicians may not disclose medical errors to patients for a variety of reasons that are related to patient's interests or physician's interests or because they may think it is useless to do so. Similarly, some patients wish to be informed about medical errors and some do not. Disclosing medical errors to patients is an issue separate from disclosing them to hospital administration. In this study we are interested in disclosing medical errors to patients. We would like to know your views on: 1) which medical error to be disclosed to patients, and 2) who to disclose medical errors to patients. There are three groups of statements for each of these two questions. The first is on what you personally prefer, the second is on what you think is best in general, and the last is on what you think reflect the current practice at KFSH&RC". For each questionnaire, participants were asked to choose the most representative statement. The six questionnaires are available in Additional file 1.

**Table 1:** Questionnaires on Personal Preference of Which Medical Error to be Disclosed and Who to Disclose it

a. Which Error to be disclosed?	
**1**	I prefer not to be informed about any medical error that occurred during my medical care.
**2**	I prefer to be informed about a medical error that occurred during my medical care if it caused a major harm (e.g. performing an unnecessary surgery).
**3**	I prefer to be informed about a medical error that occurred during my medical care if it caused at least a moderate harm (e.g. performing an unnecessary lumber puncture).
**4**	I prefer to be informed about a medical error that occurred during my medical care if it caused any harm, even a minor one (e.g. drawing an unnecessary blood sample).
**5**	I prefer to be informed about all medical error that occurred during my medical care even if it did not cause any harm (e.g. a physician orders the wrong medication but the pharmacist doesn't dispense it).
**b. Who to Disclose Error?**	
**1**	Any employee in the hospital can inform me about the medical error that occurred to me.
**2**	Any physician in the hospital can inform me about the medical error that occurred to me.
**3**	I prefer that the physician who committed the medical error informs me about the medical error that occurred to me.
**4**	I prefer that the direct manager of the physician who committed the medical error informs me about the medical error that occurred to me.
**5**	I prefer that the medical director of the hospital informs me about the medical error that occurred to me.
**6**	I prefer that the chief executive director of the hospital informs me about the medical error that occurred to me.

Eligibility criteria included age ≥18 years, ability to understand the study and provide verbal consent, and being an Arab (having nationality of one of the Arab League States). 1069 individuals in waiting areas of the outpatient clinics of KFSH&RC were invited to participate in the study. The number of individuals invited from each area was prorated based on average clinic load. The questionnaires were self or investigator administered in Arabic, according to respondent's reported educational level (≥ college education) and expressed need for assistance.

The response rate was calculated as the number of usable questionnaires (902) divided by the number of individuals approached. Data were verified by double entry. The number (% and 95% confidence interval) of respondents who chose each of the statements was determined. We used Chi^2 ^test to examine the null hypothesis of random distribution of statements' choice for each questionnaire and Kendall's W test to compare the distribution of statements' choice among the three questionnaires in each set followed by pair wise comparison by Wilcoxon Signed Ranks test. We used the Mann-Whitney test to examine if responses differed according to gender, nationality, or health status (having a chronic illness or healthy), and the Kruskal-Wallis test to examine if they differed according to education level (illiterate, school, ≥college). Correlation between age and responses to each of the three questionnaires on which ME to be disclosed was studied using Spearman's test.

We dichotomized responses to the questionnaires on which ME to be disclosed as statement one (don't disclose ME) vs. other statements, and statement 5 (disclose near miss ME) vs. other statements; and to the questionnaires on who to disclose ME as statement three (at-fault-physician) vs. other statements. The association with gender, nationality, or health status was studied using Mantel-Haenszel common odds ratio estimate. The t test was used to compare mean age. The association between five demographic variables (age, gender, nationality, health status, and educational level) and the dichotomously coded variables was modeled using forward stepwise logistic regression analysis; the odds ratios and 95% confidence intervals were estimated. Model-based means and percentages were determined by setting other variables in the model to their mean values. Analyses were conducted by one of the author (MMH) with SPSS for Windows software (release 17.0.0, 2008. SPSS Inc., Chicago, ILL, USA). 2-tailed P values are reported.

## Results

1069 individuals were approached; 63 refused to participate, 14 did not understand the study, and 90 did not complete the questionnaires. Thus responses from 902 (84%) individuals were available for analysis. The demographics of respondents are shown in Table 2.

**Table 2:** Characteristics of Study Participants (no. = 902)

**Age-mean (SD), yr**	33.9 (10)
**Gender-no. (%)**	
Male	425 (47)
Female	477(53)
**Nationality-no. (%)**	
Saudi	810 (90)
Non-Saudi Arabs*	92 (10)
**Education Level-no. (%)**	
Illiterate	46 (5)
Primary School	30 (3)
Intermediate School	75 (8)
Secondary School	199 (22)
College	159 (18)
University	392 (43)
**Occupation-no. (%)**	
Employed	438 (49)
Student	142 (16)
Housewife	267 (30)
Unemployed	54 (6)
**Chronic Illness-no. (%)**	
Present	333 (37)
Not present	569 (63)

*Egyptian, Syrian, Lebanese, Yemeni, Sudanese.

### Which medical error to disclose to patients?

As shown in Table 3, 60.0% (95% confidence interval, 56.8% to 63.2%) of respondents preferred to be informed of harmful (major, moderate, or minor) ME. In addition, 35.5% (32.4% to 38.6%) preferred to be informed of near miss ME. Only 4.5% (3.2% to 5.9%) preferred not to be informed of ME. The distribution of norm perception was not statistically different from the distribution of preference (P = 0.15). Further, there was significant correlation between the distributions of preference and norm perception (rho 0.64, P < 0.001).

**Table 3:** Which Medical Error to be Disclosed to Patients According to Personal Preference and Perceptions of Norm and Current Practice

		Disclose medical error if there is			
		
	Don't disclose	Major harm	Moderate harm	Minor harm	Near miss
**Preference **[902]	41 (4.5)	193 (21.4)	143 (15.9)	205 (22.7)	320 (35.5)
**Perception of Norm **[902]	26 (2.9)	189 (21.0)	193 (21.4)	220 (24.4)	274 (30.4)
**Perception of Practice **[846]	124 (14.7)	250 (29.6)	185 (21.9)	141 (16.7)	146 (17.3)

"Don't disclose", and disclose when there is a "major harm", "moderate harm", "minor harm", or "near miss" correspond, respectively, to statements 1 to 5 in Table 1(a). Numbers between [·] represent the number of responses. Data indicate the number (%) of respondents who chose the corresponding statement. Chi square test for the null hypothesis of random distribution was significant (P < 0.001) for each of the three questionnaires. Kendall's W coefficient of concordance (comparing the choices in the 3 questionnaires) was 0.089 (P < 0.001). Wilcoxon signed ranks test: preference vs. perception of norm, P = 0.15; preference vs. perception of current practice, p < 0.001; perception of norm vs. perception of current practice, P < 0.001.

### Who should disclose medical error to patients?

As shown in Table 4, 59.7% (56.5% to 63.0%) of respondents preferred to be informed of ME by at-fault-physician. The distribution of the norm perception was not statistically different from the distribution of preference (P = 0.33).

**Table 4:** Who to Disclose Medical Error to Patients According to Personal Preference and Perceptions of Norm and Current Practice

	Any employee	Any physician	At-fault-physician	Manager of at-fault-physician	Medical director	Chief executive director
**Preference **[867]	16 (1.8)	105 (12.1)	518 (59.7)	133 (15.3)	54 (6.2)	41 (4.7)
**Perception of Norm **[878]	25 (2.8)	120 (13.7)	502 (57.2)	147 (16.7)	46 (5.2)	38 (4.3)
**Perception of Practice **[758]	74 (9.8)	238 (31.4)	258 (34.0)	99 (13.1)	43 (5.7)	46 (6.1)

"Any employee", "any physician", "at-fault-physician", "manager of at-fault-physician", "medical director", and "chief executive director" correspond, respectively, to statements 1 to 6 in Table 1(b). Numbers between [·] represent the number of responses. Data indicate the number (%) of respondents who chose the corresponding statement. Chi square test for the null hypothesis of random distribution was significant (P < 0.001) for each of the three questionnaires. Kendall's W coefficient of concordance (comparing the choices in the 3 questionnaires) was 0.075 (P < 0.001). Wilcoxon signed ranks test: preference vs. perception of norm, P = 0.33; preference vs. perception of current practice, p < 0.001; perception of norm vs. perception of current practice, P < 0.001.

### Is there a difference between perceptions of norms and current practice?

There was significant difference (P < 0.001) between the distributions of norm perception and current practice perception in regard to which ME to be disclosed (Table 3). The distribution of current practice perception was relatively shifted to the left (i.e., less demanding). While only 2.9% (1.8% to 4.0%) perceived it as norm not to be informed of ME, 14.7% (12.3% to 17.0%) perceived that this is currently practiced. In contrast, while 30.4% (27.4% to 33.4%) perceived it as norm to be informed of near miss ME, only 17.3% (14.7% to 19.8%) perceived that this is currently practiced. Nevertheless, there was significant correlation between norm perception and current practice perception (rho 0.17, P < 0.001).

There was also significant (P < 0.001) difference between the distributions of norm perception and current practice perception regarding who to disclose ME (Table 4). The distribution of current practice perception was relatively shifted to the left (i.e., less demanding). 57.2% (53.9% to 60.5%) perceived it as norm to be informed of ME by at-fault-physician but only 34.0% (30.7 to 37.4%) perceived that this is currently practiced.

### Association between responses and respondents' demographics

As shown in Tables 5 and 6, gender was significantly associated with preference and with current practice perception. The odds ratio (OR) of male/female was 0.45 (0.34 to 0.60) for preference of disclosure of near miss ME, 4.23 (2.00 to 8.98) for preference of no-disclosure of ME, 0.40 (0.30 to 0.52) for disclosure of ME by at-fault-physician, 0.38 (0.25 to 0.57) for current practice perception that ME are not disclosed, and 0.45 (0.33 to 0.61) for current practice perception that ME are disclosed by at-fault-physician. Thus compared to males, a higher percentage of females preferred disclosure by at-fault-physician (70% vs. 48%), disclosure of near miss ME (44% vs. 26%), and a smaller percentage preferred no-disclosure of ME (2% vs. 8%). This difference between females and males in preference was consistent with their current practice perception that at-fault-physician discloses ME (43% vs. 26%) but not with which ME to be disclosed (17% vs. 17% for near miss ME, 20% vs. 9% for no-disclosure of ME); thus females had a larger gap between preference and current practice perception regarding which ME to be disclosed.

**Table 5:** Medical Error (ME) to be Disclosed According to Preference and Perception of Current Practice per Demographic Characteristics

	Statement*					**Overall P value **^**a**^	**Disclose near miss ME vs. other choices **^**b**^		Don't disclose ME **vs. other choices **^**b**^	
	Don't disclose ME	Disclose major harm ME	Disclose moderate harm ME	Disclose minor harm ME	Disclose near miss ME		P	OR (95% CI)	P	OR (95 %CI)

**Personal Preference**										

Males [425]	32 (8)	74 (17)	87 (21)	121(29)	111(26)	< 0.001	< 0.001	0.45	< 0.001	4.23
Females** [477]	9 (2)	119 (25)	56 (12)	84 (18)	209 (44)			(0.34-0.60)		(2.00-8.98)
Saudis [810]	38 (5)	182 (23)	129 (16)	176 (22)	285 (35)	0.06	0.59	0.88	0.54	1.46
Non-Saudi Arabs** [92]	3 (3)	11 (12)	14 (15)	29 (32)	35 (38)			(0.57-1.4)		(0.44-4.83)
Patients [333]	8 (2)	80 (24)	57 (17)	84 (25)	104 (31)	0.25	0.04	0.74	0.02	0.40
Healthy** [569]	33 (6)	113 (20)	86 (15)	121 (21)	216 (38)			(0.56-0.99)		(0.18-0.88)
≥College, 551	21 (4)	114 (21)	101 (18)	128 (23)	187 (34)	0.02	0.003	0.40	0.58	1.78
								(0.21-0.73)		(0.23-13.51)
School [304]	19 (6)	70 (23)	42 (14)	67 (22)	106 (35)		0.006	0.41	0.29	3.00
								(0.22-0.77)		(0.39-22.73)
Illiterate** [46]	1 (2)	9 (20)	0 (0)	10 (22)	26 (57)					

**Perception of Current Practice**										

Males [420]	37 (9)	104 (25)	119 (28)	87 (21)	73 (17)	< 0.001	0.93	1.02	< 0.001	0.38
Females** [426]	87 (20)	146 (34)	66 (16)	54 (13)	73 (17)			(0.71-1.45)		(0.25-0.57)
Saudis [758]	116 (15)	230 (30)	161 (21)	125 (17)	126 (17)	0.02	0.15	0.68	0.12	1.81
Non-Saudi Arabs** [88]	8 (9)	20 (23)	24 (27)	16 (18)	20 (23)			(0.40-1.16)		(0.85-3.84)
Patients [304]	41 (14)	88 (29)	60 (20)	59 (19)	56 (18)	0.20	0.50	1.13	0.47	0.86
Healthy** [542]	83 (15)	162 (30)	125 (23)	82 (15)	90 (17)			(0.79-1.64)		(0.58-1.29)
≥College [524]	66(13)	167 (32)	136 (26)	73 (14)	82 (16)	0.004	0.83	1.11	0.01	0.31
								(0.42-2.95)		(0.17-0.78)
School [286]	48 (17)	66 (23)	49 (17)	64 (22)	59 (21)		0.38	1.56	0.09	0.50
								(0.58-4.20)		(0.23-1.12)
Illiterate** [35]	10 (29)	16 (46)	0 (0)	4 (11)	5 (14)					

*"Don't disclose", and disclose when there is a "major harm", "moderate harm", "minor harm", or "near miss" correspond, respectively, to statements 1 to 5 in Table 1(a). Data indicate the number (%) of respondents who chose the corresponding statement. Numbers between [·] represent the number of responses from participants with the indicated characteristic. ^a^Mann-Whitney test or Kruskal-Wallis test (for educational status). ^b^Mantel-Haenszel common odds ratio estimate.

** Reference group in the Mantel-Haenszel estimate. Percentages may not add up to 100 due to rounding.

**Table 6:** Who to disclose Medical Error According to Public Preference and Perception of Current Practice per Demographic Characteristics

	Statement*						**Overall P **^**a**^	**At-fault-physician vs. other choices **^**b**^	
	Any employee	Any physician	At-fault physician	Manager of At-fault-physician	Medical director	Chief executive director		P	OR (95% CI)

**Personal Preference**									

Males [399]	7 (2)	65 (16)	191 (48)	77 (19)	36 (9)	23 (6)	0.04	< 0.001	0.40
Females** [468]	9 (2)	40 (9)	327 (70)	56 (12)	18 (4)	18 (4)			(0.30-0.52)
Saudis [778]	14 (2)	92 (12)	468 (60)	117(15)	53 (7)	34 (4)	0.74	0.50	1.18
Non-Saudi Arabs** [89]	2 (2)	13 (15)	50 (56)	16 (18)	1 (1)	7 (8)			(0.76-1.83)
Patients [327]	8 (2)	44 (14)	202 (62)	48 (15)	16 (5)	9 (3)	0.02	0.34	1.15
Healthy** [540]	8 (2)	61 (11)	316 (59)	85 (16)	38 (7)	32 (6)			(0.87-1.52)
≥College [534]	11 (2)	68 (13)	310 (58)	95 (18)	32 (6)	18 (3)	0.10	0.13	0.61
									(0.32-1.16)
School [286]	3 (1)	31 (11)	176 (62)	34 (12)	21 (7)	21 (7)		0.30	0.70
									(0.36-1.37)
Illiterate** [46]	2 (4)	6 (13)	32 (70)	4 (9)	1 (2)	1 (2)			

**Perception of Current Practice**									

Males [405)	54 (13)	118 (29)	104 (26)	76 (19)	27 (7)	26 (6)	0.50	< 0.001	0.45
Females** [353]	20 (6)	120 (34)	154 (43)	23 (7)	16 (5)	20 (6)			(0.33-0.61)
Saudis [674]	66 (10)	215 (32)	229 (34)	87 (13)	40 (6)	37 (6)	0.32	0.92	0.98
Non-Saudi Arabs** [84]	8 (10)	23 (27)	29 (35)	12 (14)	3 (4)	9 (11)			(0.61-1.57)
Patients [270]	14 (5)	91 (34)	102 (38)	38 (14)	15 (6)	10 (4)	0.48	0.11	1.29
Healthy** [488]	60 (12)	147 (30)	156 (32)	61 (13)	28 (6)	36 (7)			(0.95-1.76)
≥College [478]	58 (12)	158 (33)	154 (32)	61 (13)	25 (5)	22 (5)	0.002	0.42	0.60
									(0.27-1.36)
School [254]	15 (6)	70 (28)	93 (37)	36 (14)	17 (7)	23 (9)		0.47	0.74
									(0.32-1.69)
Illiterate** [25]	1 (4)	10 (40)	11 (44)	1 (4)	1 (4)	1 (4)			

*"Any employee", "any physician", "at-fault-physician", "manager of at-fault-physician", "medical director", and "chief executive director" correspond, respectively, to statements 1 to 6 in Table 1(b). Data indicate the number (%) of respondents who chose the corresponding statement. Numbers between [·] represent the number of responses from participants with the indicated characteristic. ^a^Mann-Whitney test or Kruskal-Wallis test (for educational status). ^b^Mantel-Haenszel common odds ratio estimate.

** Reference group in the Mantel-Haenszel estimate. Percentages may not add up to 100 due to rounding.

Nationality (Saudis vs. Non-Saudi Arabs) was not significantly associated with preference or perception. Health status was significantly associated with preference of which ME to be disclosed. The OR (patient to healthy) was 0.74 (0.56 to 0.99) for preference of disclosure of near miss ME and 0.40 (0.18 to 0.88) for preference of no-disclosure of ME, although the overall distributions of preferences were not statistically different Thus patients were less likely than healthy individuals to prefer the more extreme choices. Current practice perceptions of patients and healthy individuals were not statistically different (Table 5 &6).

Education was significantly associated with preference and current practice perception of disclosure of ME. A higher percentage of illiterates preferred to be informed of near miss ME and perceived that in current practice ME are not disclosed. The OR of school graduates and ≥ college graduates to illiterates was 0.41 (0.22 to 0.77) and 0.40 (0.21 to 0.73), respectively, for preference of disclosure of near miss ME and 0.50 (0.23 to 1.12) and 0.31 (0.17 to 0.78) for the perception that under current practice ME are not disclosed (Table 5). Twenty nine percent of the illiterates, 17% of school graduates, and 13% of ≥college graduates perceived that current practice doesn't disclose ME, whereas 2%, 6%, and 4%, respectively, preferred such practice, indicating a larger gap between preference and perception of current practice with lower education level (Table 5).

Age was significantly associated with preference. Individuals who preferred to be informed of near miss ME or who preferred to be informed by at-fault-physician were older than the rest with a mean (95% confidence interval) difference of 1.60 (0.23 to 2.30) year (P = 0.02) and 1.65 (0.29 to 3.01) year (P = 0.02), respectively, whereas individuals who preferred not to be informed of ME were younger with a mean difference of 4.36 (1.22 to 7.51) year (P = 0.007). There was no significant association between age and current practice perception regarding disclosure of near miss ME, no-disclosure of ME, or disclosure by at-fault-physician (P = 0.83, P = 0.19, P = 0.76, respectively, for mean age difference). Further, age correlated positively with preference score (statement number, 1 to 5) of which ME to be disclosed (rho 0.11, P = 0.001) but not with current practice score (rho -0.03, P = 0.45).

We used a forward stepwise logistic regression model that included age, gender (female as reference group), nationality (Non-Saudi Arabs as a reference group), health status (healthy as a reference group), and educational level (illiterates as reference group). For predicting preference of disclosure of near miss ME (vs. other preferences), only age, gender, and health status remained in the model as significant predictors (OR 1.02 (1.01 to 1.04) per year, P = 0.008; 0.45 (0.34 to 0.60), P < 0.001; and 0.60 (0.44 to 0.82), P = 0.001, respectively). For predicting preference of no-disclosure of ME (vs. other preferences), only age and gender were significant predictors (OR 0.96 (0.92 to 0.99) per year, P = 0.02 and 3.95 (1.86 to 8.39), P < 0.001, respectively). There were no significant predictors of the perception that in current practice patients are informed of near miss ME; and only gender predicted the perception that patients are not informed of ME (OR 0.38 (0.25 to 0.57), P < 0.001). The logistic regression model also showed that gender predicted preference of ME disclosure by at-fault-physician and the perception that in current practice ME are disclosed by at-fault-physician (OR 0.49 (0.35 to 0.69), P < 0.001 and 0.45 (0.33 to 0.61), P < 0.001, respectively).

## Discussion

The aim of this study was to survey public views on disclosure of ME to patients in the outpatient setting at a tertiary healthcare center in Riyadh, Saudi Arabia. We studied a convenient sample of 902 adult Arabs and examined three perspectives (preference, perception of norm, and perception of current practice) on two topics (which ME to disclose and who to disclose ME). The study sample had a mean (SD) age of 33.9 (10) year, 47% males, 90% Saudis, 37% patients with chronic illness (63% were independent healthy patients' companions), 49% employed, and 61% with college or higher education. The strengths of the study include a large sample size, a high response rate, simultaneous examination of preference, perception of norm, and perception of current practice, specifically addressing near miss ME, and being the first in the Islamic/Arab culture. Its weaknesses include that it was performed in a single institution and thus the results may not be generalizable, and that it did not compare people who were or were not actually exposed to ME. We found that: 1) there is a considerable diversity in preferences and perceptions of norm and current practice among respondents both regarding which ME to disclose and by whom, 2) distributions of preference and perception of norm were similar but significantly different from the distribution of perception of current practice, 3) most respondents preferred to be informed of ME and by at-fault-physician, and 4) one third of respondents preferred to be informed of near miss ME, with a higher percentage among females, older, and healthy individuals.

The observed diversity in preferences suggests that a one-fits-all policy on ME disclosure may results in patient's dissatisfaction. The diversity in perceptions of norm may be due to an absence of a norm or that the norm is not well known to the public. KFSH&RC has been accredited by the Joint Commission on International Accreditations since November 2000, and ME disclosure is included in its policy. However, the Rules of Implementation for Regulation of the Practice of Medicine and Dentistry that were promulgated by the Saudi Ministry of Health in January 1990 [34] and the more recent Ethics of Medical Profession released by the Saudi Commission of Health Specialists are silent on the issue [35]. From Islamic teachings point of view, it is expected that a mistake or error is disclosed, an apology is given, and forgiveness is sought. Prophet Muhammad has taught, "Whoever has oppressed another person concerning his reputation or anything else, he should beg him to forgive him before the Day of Resurrection when there will be no money (to compensate for wrong deeds), but if he has good deeds, those good deeds will be taken from him according to his oppression which he has done, and if he has no good deeds, the sins of the oppressed person will be loaded on him."[36]. This would be understood to imply that the Islamic norm is to disclose, sincerely apologize, and rectify all harmful ME, even if associated with only a minor harm. Interestingly, in a physicians' survey, respondents who agreed that forgiveness is important for their spiritual or religious belief were more likely to disclose a hypothetical error resulting in a minor harm [37].

Our failure to find significant differences between preferences and perceptions of norm may be due to respondents' inability to differentiate between the two. This is not likely because they were relatively highly educated (61% had college or higher education) and the two questions were presented at the same time. Alternatively, it may reflect a rather norm-desiring culture that seeks harmony between motives (preference) and reasons (perception of norm) or a social desirability bias (a low inclination to express a preference that is different from the perceived norm).

We found that the distribution of perception of current practice regarding which ME to disclose is shifted to the left (less demanding) compared to the distributions of preference and perception of norm. Relatively, more disclosure was preferred and perceived as norm, indicating a degree of disagreement and dissatisfaction of the public with current practice. Respondents were asked about their perception of ME disclosure practice at the KFSHRC, a leading hospital in the area. A larger disclosure gap would be expected for other hospitals. Although patients may be mistaken in their perception of current practice, such gap has been well documented in previous studies under different settings, and has been attributed to the difference between patients' and physicians' declared preferences and between physicians' preference and what they actually do [5,10,37,38]. Another potential contributor to this gap is respondents' inclusion of unanticipated outcomes among ME. In a study in the rural areas in USA, 41% of the ME perceived by 172 respondents involved only unanticipated outcome [39]. Patients may have a broader definition of ME that includes poor service quality, significant delay in treatment, non-preventable adverse events, and deficient interpersonal skills [5]. A community survey in Oman found that only 78% of participants believed they knew what was meant by ME [40]. The discrepancy between preference and perception of current practice in our study was mainly in extreme choices (no-disclosure and disclosure of near miss ME) and more pronounced among females and people with lower education. This may indicate more dissatisfaction with current practice in these two groups. A previous study did not reveal an association between preference for ME disclosure and gender or education level [14].

Consistent with previous studies [5,10,13,14,17], we found that overall 95.5% of respondents preferred to be informed of ME. It is not known if this consistently expressed preference would change during an acute illness [10,41]. Interestingly, compared to healthy individuals, patients in our study were more likely to prefer disclosure of harmful ME and less likely to prefer the more extreme choices (no-disclosure and disclosure of near miss ME).

Current regulations are silent regarding disclosure of near miss ME and while some authorities recommend disclosure [21-24], others disagree [[2,25], and [26]]. An important finding of our study is that 35.5% of respondents preferred to be informed of near miss ME. This is a much smaller percentage than the 88-92% reported in previous studies [15,27]. Interestingly, our study also showed that 30.4% of respondents perceived that the norm is to disclose near miss ME. Do patients have the ethical right to be informed of near miss ME? Although reporting of near miss ME is an important quality improvement measure [1,3] and public disclosure of near miss ME (in aggregate) can be considered part of informed consent for future patients, it is arguable that one can defend disclosure of near miss ME to the patient involved based on the Rights approach or Rule-Utilitarian approach (looks at the consequences of having everyone follow a particular rule and calculates the overall utility of accepting or rejecting the rule) to ethics [42]. On one hand, one can argue that as an extension of the principle of autonomy, patients have the right to know of near miss ME because it may help them make more informed healthcare choices, alert them to which error they should watch for, and create an opportunity for them to be part of quality improvement efforts [5]. It would also reassure them that the systems to prevent ME from reaching them were working [5], educate them about the uncertainty of medicine, promote faith in the patient/physician relationship, provide a check point in case the healthcare system fails to improve, and allow gaining absolution from truth telling and confession on part of physicians [42]. Two third of physicians believed that disclosing a mistake to the patient would help alleviate their feeling of guilt [37] and 74% of whoever disclosed a serious error experienced a relief after disclosure [45]. On the other hand, disclosure of near miss ME may raise in the patient feelings of anger, "useless" dissatisfaction, and undue suspicion. Physicians may have uncertainties about patient's response to disclosure, whether an error actually occurred, and how to disclose it, and may feel a sense of shame - I was what was wrong [43] and helplessness (losing control over the situation), especially in a culture that focuses on individual responsibility rather than system improvement. Further, such disclosure may be impractical for physicians [19] and may put them at a conflict between their others-regarding posture and self-regarding posture [42] as they fear loss of reputation and malpractice litigation [37]. In this regard, a recent welcome legal development in some countries is a medical apology law that protects physicians who apology to patients from admission of medical liability [44]. Furthermore, near miss disclosure is not practiced outside the medical field, for example, people are not informed of human errors in maintaining or flying airplanes if harm doesn't occur, although this also involves human lives. Requiring physicians to be more virtuous than the society as a whole may not be fair or reasonable [42]. Thus disclosure of near miss ME may be better addressed through the Virtue approach to ethics (promotes situational appreciation since virtue is an activity in accordance with reason), ethical relativism (morality is culture- and circumstance-dependent), and Act-Utilitarianism (looks at the consequences of each individual act and calculate utility each time the act is performed). This is consistent with the recommendations of the American Society for Reproductive Medicine [26]. In our study, females, older peoples, and healthy individuals were significantly more likely to indicate preference for disclosure of near miss ME. It is not clear why some people are more likely to be interested in information that may be disappointing and have no practical use. It could be due to a higher desire for information in general, relative lack of other competing information, or a higher degree of autonomy. This has not been addressed in previous studies. There were no significant differences among the various demographic groups in current practice perception of disclosure of near miss ME.

Some believe that ME disclosure must be conducted as mush as possible by those originally involved in patient care [10,11,22,29] and that physicians should take responsibility for their own errors by personally disclosing and apologizing for them [46]. Others have advised that the healthcare provider "who last touched the patient" should not be part of the disclosure team, at least initially, because he/she may be lacking the required communication skills and experiencing emotional turmoil [21,28]. Thirty four percent of our respondents (43% of females and 26% of males) have the perception that under current practice ME are disclosed by at-fault-physician. A US national survey showed that only 30% of the respondents who experienced ME were reportedly informed by the involved healthcare provider [47]. Almost 60% of respondents (70% of females and 48% of males) in the current study preferred and perceived it as norm to be informed of ME by at-fault-physician. This may indicate that they want an apology rather than a disciplinary action or just information and that they see ME as a physician responsibility rather than an organizational/systems issue. Understanding cultural expectations such as truth telling and forgiveness can provide insight into patients needs [46]. Just as secular Western societies continue to be influenced by Judo-Christian norms concerning social ethics [46], Arabic and Islamic societies are still influenced by Islamic social ethics which shares many foundational values with Judaism and Christianity [48]. Forgiveness and truth telling are praised in several verses of Quran, for example: "Be quick in the race for forgiveness from your Lord, and for a Garden whose width is that (of the whole) of the heavens and of the earth, prepared for the righteous. Those who spend (freely), whether in prosperity, or in adversity; who restrain anger, and pardon (all) men; for Allah loves those who do good." (Chapter 3, verses 133,134), "O ye who believe! stand out firmly for justice, as witnesses to Allah, even as against yourselves, or your parents, or your kin, and whether it be (against) rich or poor: for Allah can best protect both. Follow not the lusts (of your hearts), lest ye swerve, and if ye distort (justice) or decline to do justice, verily Allah is well-acquainted with all that ye do."(Chapter 4, verse 135) [49]. Fulfilling patients' cultural expectations may help patients forgive physicians and physicians reach self-forgiveness; a systems approach may not apply here since the patient/physician relationship is perceived to be present between individuals, not between a person and a "system" [46]. An apology for ME implies admission of fault and expression of regret for the action and sympathy for the results, but does not necessarily contain an acknowledgment of responsibility [44]. At a cognitive level, apology allows patients to take the perspective of the "at-fault-physician", having a more positive perception of his/her character and recognizing that the circumstances may have played a role. At an affective level, it allows patients to appreciate that the "at-fault-physician" is also suffering [44]. The type of apology, whether an apology of regret/sympathy or an apology of responsibility, may not be as important in soothing patient's anger and suspicion as the perceived sincerity [10,50], and this can be best conveyed by the at-fault-physician.

## Conclusions

In the setting of outpatient clinics at a tertiary care hospital in Saudi Arabia, we found that: 1) There is a considerable diversity in preferences and perceptions of norm and current practice regarding which ME to disclose and by whom, which may indicate that a one-fits-all policy for ME disclosure may result in patients' dissatisfaction and that there is a need for more public education on patients' rights regarding ME disclosure. 2) Distributions of preference and perception of norm were similar, suggesting a rather norm-desiring culture, but significantly different from the distribution of perception of current practice, suggesting a perceived gap between preference/norm and current practice, especially in females and in individuals with lower education level. 3) Most respondents preferred to be informed of ME, consistent with previous studies in other cultures, and to be informed by at-fault physician, indicating that they are looking for an apology rather than a disciplinary action or just information. Finally, only one third of respondents preferred to be informed of near miss ME, which is much lower than what was reported in previous studies, with even a lower percentage among males, young, and sick individuals.

## Competing interests

The authors declare that they have no competing interests.

## Authors' contributions

MMH conceived of the study, participated in study design, performed statistical analysis, participated in literature review, and wrote the manuscript. SA participated in data collection, literature review, and statistical analysis. MA participated in study design, data collection, and literature review. All authors read and approved the final manuscript.

## Pre-publication history

The pre-publication history for this paper can be accessed here:

http://www.biomedcentral.com/1472-6939/11/17/prepub

## Supplementary Material

Additional file 1 **Study questionnaires**. The six study questionnaires. Click here for file
