# Supplementary material for: Which medical error to disclose to patients and by whom? Public preference and perceptions of norm and current practice
Source: BMC Med Ethics. 2010 Oct 18;11:17. doi: 10.1186/1472-6939-11-17 (PMC2967555; doi:10.1186/1472-6939-11-17)
Supplement: Additional file 1 — Study questionnaires. The six study questionnaires. [file 1472-6939-11-17-S1.DOC]

**Dear participant:**

Clinical practice, just like any other beneficial practice, could hardly be completely free from harm. Such harm can be divided into two types: 1) harm that can be predicted and thus can be avoided, e.g. anaphylactic shock caused by penicillin administration to a person known to have penicillin allergy, and 2) harm that can’t be predicted/avoided, e.g. inflammation of the bowel after some antibiotics treatment. The first type is called medical error. A medical error is defined as the failure to complete planned medical action as intended, or the use of a wrong plan to achieve an aim. Medical errors may or may not cause harm, for example, penicillin could be wrongly prescribed by a physician but not given to the patient because the error is discovered and corrected in time by a pharmacist or nurse.

Physicians may not disclose medical errors to patients for a variety of reasons that are related to patient’s interests or physician’s interests or because they may think it is useless to do so. Similarly, some patients wish to be informed about medical errors and some do not. Disclosing medical errors to patients is an issue separate from reporting them to hospital administration. In this study we are interested in disclosing medical errors to patients.

We would like to know your views on: 1) which medical error to be disclosed to patients, and 2) who to disclose medical errors to patients. There are three groups of statements for each of these two questions. The first is on what you personally prefer, the second is on what you think is best in general and the last is on what you think reflect the current practice at KFSH&RC.

**Part I: Personal Preference (what I personally prefer)**

a) Which medical error to be disclosed? (Please choose one answer.)

1) I prefer not be informed about any medical error that occurred during my medical care.

2) I prefer to be informed about a medical error that occurred during my medical care if it caused a major harm (e.g. performing an unnecessary surgery).

3) I prefer to be informed about a medical error that occurred during my medical care if it caused at least a moderate harm (e.g. performing an unnecessary lumber puncture).

4) I prefer to be informed about a medical error that occurred during my medical care if it caused any harm, even a minor one (e.g. drawing an unnecessary blood sample).

5) I prefer to be informed about a medical error that occurred during my medical care even if it did not cause any harm (e.g. a physician orders the wrong medication but the pharmacist doesn’t dispense it).

b) Who to disclose medical errors? (Please choose one answer.)

1) Any employee in the hospital can inform me about the medical error that occurred to me.

2) Any physician in the hospital can inform me about the medical error that occurred to me.

3) I prefer that the physician who committed the medical error informs me about the medical error that occurred to me.

4) I prefer that the direct manager of the physician who committed the medical error informs me about the medical error that occurred to me.

5) I prefer that the medical director of the hospital informs me about the medical error that occurred to me.

6) I prefer that the chief executive director of the hospital informs me about the medical error that occurred to me.

**Part II: What I think is appropriate in general regardless of what I personally prefer**

a) Which medical error to be disclosed? (Please choose one answer.)

1) I think that the patient should not be informed of any medical error that occurred during his medical care.

2) I think that the patient should be informed of a medical error that occurred during his medical care if it caused a major harm (e.g. performing an unnecessary surgery).

3) I think that the patient should be informed of a medical error that occurred during his medical care if it caused at least a moderate harm (e.g. performing an unnecessary lumber puncture).

4) I think that the patient should be informed of a medical error that occurred during his medical care if it caused any harm, even a minor one (e.g. drawing an unnecessary blood sample).

5) I think that the patient should be informed of a medical error that occurred during his medical care even if it did not cause any harm (e.g. a physician orders the wrong medication but the pharmacist doesn’t dispense it).

b) Who to disclose medical errors? (Please choose one answer.)

1) Any employee in the hospital can inform the patient about the medical error that occurred to him.

2) Any physician in the hospital can inform the patient about the medical error that occurred to him.

3) I think that the physician who committed the medical error should inform the patient about the medical error that occurred to him.

4) I think that the direct manager of the physician who committed the medical error should inform the patient about the medical error that occurred to him.

5) I think that the medical director of the hospital should inform the patient about the medical error that occurred to him.

6) I think that the chief executive director of the hospital should inform the patient about the medical error that occurred to him.

**Part III: What I think reflects current practice at KFSH&RC regardless of what I personally prefer or think is appropriate in general**

a) Which medical error to be disclosed? (Please choose one answer.)

1) The patient is not informed of any medical error that occurred during his medical care.

2) The patient is informed of a medical error that occurred during his medical care if it caused a major harm (e.g. performing an unnecessary surgery).

3) The patient is informed of a medical error that occurred during his medical care if it caused at least a moderate harm (e.g. performing an unnecessary lumber puncture).

4) The patient is informed of a medical error that occurred during his medical care if it caused any harm, even a minor one (e.g. drawing an unnecessary blood sample draw).

5) The patient is informed of a medical error that occurred during his medical care even if it did not cause any harm (e.g. a physician orders the wrong medication but the pharmacist doesn’t dispense it).

b) Who to disclose medical errors? (Please choose one answer.)

1) Any employee in the hospital informs the patient about the medical error that occurred to him.

2) Any physician in the hospital informs the patient about the medical error that occurred to him.

3) The physician who committed the medical error informs the patient about the medical error that occurred to him.

4) The direct manager of the physician who committed the medical error informs the patient about the medical error that occurred to him.

5) The medical director of the hospital informs the patient about the medical error that occurred to him.

6) The chief executive director of the hospital informs the patient about the medical error that occurred to him.
